# Pilot study of an interprofessional pediatric mechanical ventilation educational initiative in two intensive care units

**DOI:** 10.1186/s12909-023-04599-1

**Published:** 2023-08-28

**Authors:** Pazun Mehrzai, Thormen Höfeler, Chinedu Ulrich Ebenebe, Parisa Moll-Khosrawi, Süha Demirakça, Eik Vettorazzi, Marlies Bergers, Mandy Lange, Sabine Dreger, Hanna Maruhn, Dominique Singer, Philipp Deindl

**Affiliations:** 1 Department of Neonatology and Pediatric Intensive Care Medicine, University Children’s Hospital, University Medical Center Hamburg-Eppendorf, Martinistr. 52, Hamburg, 20246 Germany; 2 Department of Anesthesiology, University Medical Center Hamburg-Eppendorf, Hamburg, Germany; 3 Department of Neonatology Pediatric Intensive Care and Pulmonology, Children’s Hospital University Mannheim, Mannheim, Germany; 4 Department of Medical Biometry and Epidemiology, Institute of Medical Biometry and Epidemiology, University Medical Center Hamburg-Eppendorf, Hamburg, Germany

**Keywords:** Educational initiative, Team performance, Treatment goal compliance, Selfconfidence, Checklists, Educational film

## Abstract

**Introduction:**

Inappropriate ventilator settings, non-adherence to a lung-protective ventilation strategy, and inadequate patient monitoring during mechanical ventilation can potentially expose critically ill children to additional risks. We set out to improve team theoretical knowledge and practical skills regarding pediatric mechanical ventilation and to increase compliance with treatment goals.

**Methods:**

An educational initiative was conducted from August 2019 to July 2021 in a neonatal and pediatric intensive care unit of the University Children’s Hospital, Hamburg-Eppendorf, Germany. We tested baseline theoretical knowledge using a multiple choice theory test (TT) and practical skills using a practical skill test (PST), consisting of four sequential Objective Structured Clinical Examinations of physicians and nurses. We then implemented an educational bundle that included video self-training, checklists, pocket cards, and reevaluated team performance. Ventilators and monitor settings were randomly checked in all ventilated patients. We used a process control chart and a mixed-effects model to analyze the primary outcome.

**Results:**

A total of 47 nurses and 20 physicians underwent assessment both before and after the implementation of the initiative using TT. Additionally, 34 nurses and 20 physicians were evaluated using the PST component of the initiative. The findings revealed a significant improvement in staff performance for both TT and PST (TT: 80% [confidence interval (CI): 77.2–82.9] vs. 86% [CI: 83.1–88.0]; PST: 73% [CI: 69.7–75.5] vs. 95% [CI: 93.8–97.1]). Additionally, there was a notable increase in self-confidence among participants, and compliance with mechanical ventilation treatment goals also saw a substantial rise, increasing from 87.8% to 94.5%.

**Discussion:**

Implementing a pediatric mechanical ventilation education bundle improved theoretical knowledge and practical skills among interprofessional pediatric intensive care staff and increased treatment goal compliance in ventilated children.

**Supplementary Information:**

The online version contains supplementary material available at 10.1186/s12909-023-04599-1.

## Introduction

Managing mechanical ventilation (MV) for infants, children, and adolescents is a complex skill. MV of neonates and children is a life-saving procedure but can also lead to severe complications such as ventilator-associated lung injury [1–4]. Severely diseased lungs are especially susceptible to shear forces due to high tidal volume (TV) [5]. Lung-protective MV aims to create physiological conditions to prevent lung damage. International guidelines for neonatal and pediatric MV recommend avoidance of high TV and delta pressure (peak inspiratory pressure [PIP]—positive end-expiratory pressure [PEEP]) in acute respiratory distress syndrome [6–9]. Although few robust data are available, excessive TV and pressures may also injure healthy lung tissue in mechanically ventilated children [10–12].

In our institution, the nursing staff prepared and set up the ventilators, whereas the physicians adjusted settings according to clinical indications. The combination of this task sharing with inconsistent approaches regarding ventilator settings and the lack of specifically defined goals during MV led to inappropriate ventilator setup and settings, non-adherence to a lung-protective ventilation strategy, and inadequate patient monitoring.

In the realm of medical education, numerous clinical observation tools have been developed to evaluate the clinical skills of medical students and trainees. One notable review conducted by Kogan et al. extensively examined tools for Direct Observation and Assessment of Clinical Skills of Medical Trainees, with a particular emphasis on assessing skills related to history taking, examination, communication, and counseling [13]. The findings of the review underscore the significance of performance-based clinical skills assessment and the availability of various tools for direct observation. However, the authors highlight a noteworthy gap in the literature regarding the scarcity of validity evidence and comprehensive descriptions of educational outcomes associated with these tools. Objective Structured Clinical Examinations (OSCEs) are widely recognized and utilized as evidence-based procedures for learning and assessing practical skills. The literature supports their effectiveness in various domains, including healthcare education [14, 15]. To our knowledge, there is no published OSCE exam that specifically tests practical skills related to pediatric ventilation. O'Boyle et al. developed and validated a tool for testing theoretical knowledge of pediatric staff in mechanical ventilation [16]. In contrast, our approach involved the development of two theory tests, tailored to target the specific weaknesses and focus areas within our clinic, with particular emphasis on high-frequency ventilation and the utilization of inhaled nitric oxide.

The integration of interprofessional education (IPE) plays a pivotal role in adequately preparing physicians and nurses for their future roles in the healthcare workforce, where effective teamwork and collaboration are vital competencies. Recognizing its significance, several international health organizations have actively advocated for the implementation of IPE as a means to reshape healthcare systems, foster interprofessional teamwork, elevate the quality of patient care, and ultimately enhance health outcomes [17]. Interprofessional educational programs can improve theoretical knowledge and skills [18–21], but their effect on mechanical ventilation settings and compliance to treatment goals in actual pediatric patients remains unclear.

This interprofessional educational initiative aimed to improve team knowledge and practical skills regarding pediatric MV and to optimize compliance with treatment goals that included a lung-protective ventilation strategy. The first aim was to achieve consensus on a specification and standardization of ventilator setup, parameter settings, and a lung-protective MV strategy for all ventilated children. Therefore, we tested both nurses’ and physicians’ baseline theoretical knowledge and practical skills regarding MV in children to develop and implement a highly standardized specific educational program. The second aim was to increase theoretical knowledge and practical staff performance regarding MV. The third aim was to increase compliance with patient-specific treatment goals including a lung-protective ventilation strategy to > 90% during the twelve months after the intervention.

## Methods

We performed this educational initiative in the 12-bed Pediatric Intensive Care Unit (PICU) and the 15-bed Neonatal Intensive Care Unit (NICU) of the Level-IV University Children’s Hospital, University Medical Center Hamburg-Eppendorf, Germany, between August 2019 and July 2021 after approval of the local ethical review committee (Ethikkommission der Ärztekammer Hamburg, Germany).

**Fig. 1:**
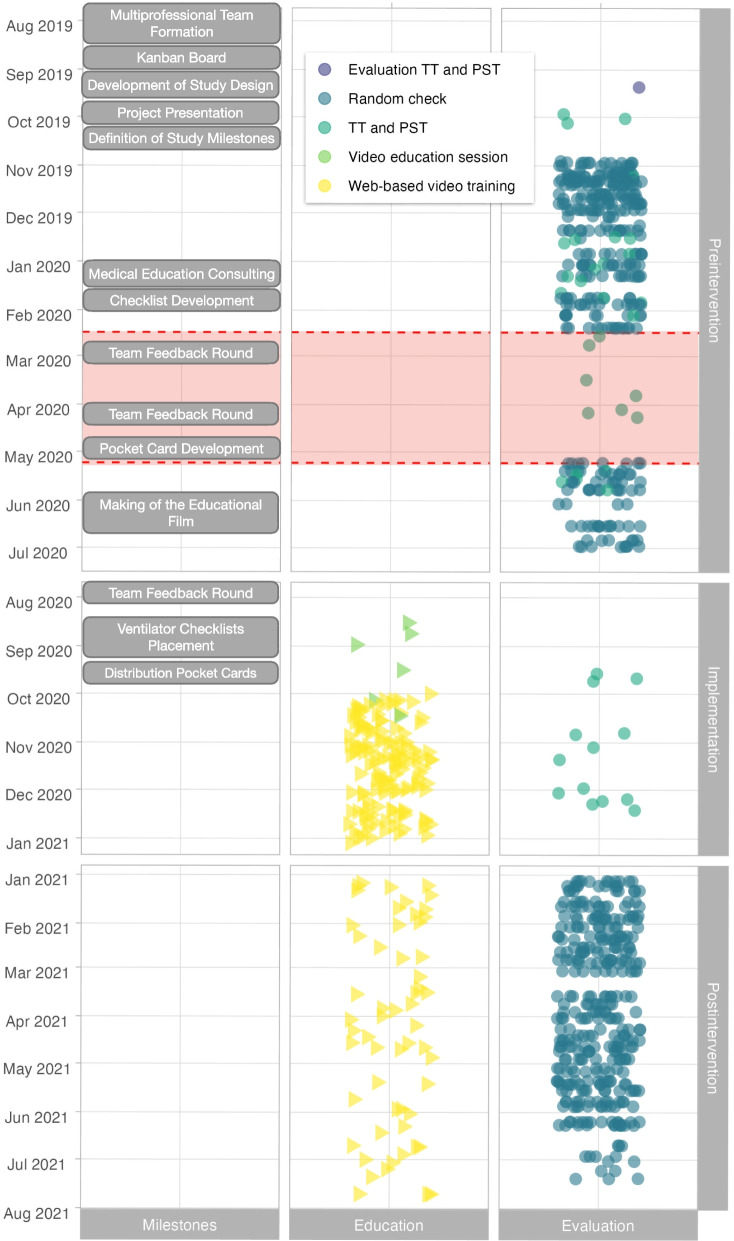
Timeline and Key Events during the Initiative to Improve Ventilation Quality in Children in Neonatal and Pediatric Intensive Care Units. This figure illustrates the chronological sequence of milestones, training, and evaluation measures during the initiative to enhance ventilator quality for children in neonatal and pediatric intensive care units. The timeline represents pre-intervention, implementation, and post-intervention time points. As part of the intervention, staff engaged in self-study by streaming the educational film 185 times (yellow triangles). Additionally, six collaborative video training sessions with discussions were conducted to enhance training (light green triangles). Team performance was assessed through theory tests (TT) and practical skill tests (PST) during 42 evaluation sessions (light green dots). Furthermore, compliance with patient-specific treatment goals was monitored through 662 random checks across 213 patients receiving respiratory support in both intensive care units (dark blue-green dots). During the SARS-CoV-2 pandemic, between February 2020 and April 2020, the random checks were temporarily paused due to visiting restrictions (light red ribbon)

An interprofessional team of physicians and nurses supervised the project. During the planning phase, we collected ideas and suggestions on potential improvements and current problems related to pediatric MV from the medical and nursing teams of both ICUs. In addition, we regularly exchanged ideas via a “Kanban Board” through surveys and regular team feedback rounds (Fig. 1). Checklists for set-up and settings according to treatment goals were fixed permanently and visibly to each ventilator. Additionally, pocket cards were distributed to all staff summarizing essential educational topics and mnemonic aids (see Supplementary Material). A team consisting of intensive care nurses, physicians, medical education specialists, and communication experts developed the instructive content for a 30-min educational film. The film covered the above-mentioned treatment goals, ventilation, oxygenation, inhaled nitric oxide (iNO), and complications of MV (www.uke.de/picu-nicu). We trained staff members by showing the film, answering questions, and discussing critical topics during training sessions in both ICUs. In addition, each staff member had access to the film from home and any hospital computer for self-study (Fig. 1).

In joint discussions and regular feedback rounds, a highly standardized approach was developed and adapted to set up ventilators and to establish specific start settings according to patient weight categories (see Supplementary Material). After several team feedback rounds during staff meetings, we expanded the contents of the checklists, pocket cards, and educational film (Fig. 1).

We developed a theory test (TT) following the specific topics of a validated testing tool for pediatric mechanical ventilation [16] and additional topics to evaluate physicians’ and nurses’ theoretical knowledge at baseline. Our approach involved close collaboration with didactic and subject matter experts to construct a question pool consisting of 30 questions. These questions were then subjected to evaluation for difficulty and stringency through a pilot test involving ten staff members. The pilot test was not included in the final analysis. The valuable feedback received from the participants in the pilot test was utilized to curate the 15 questions for each of the two theory tests covering the following topics: ventilation, oxygenation, iNO, high-frequency oscillatory ventilation (HFOV), and complications of MV, ensuring their comparability and appropriateness for the assessments (see Supplementary Material). A questionnaire recorded the participant’s profession and experience. We randomly assigned the participants to one of two TTs using a randomization list.

A practical skill test (PST), consisting of four sequential Objective Structured Clinical Examinations (OSCE) [14, 15, 21, 22] was taken directly at a modified ICU workplace. We randomly assigned the participants to one of the two equally difficult PSTs using a randomization list. Assessors observed the participants during the PST and assigned a maximum of 30 points based on performance-structured checklists (see Supplementary Material). The PST simulated four clinical commonly encountered challenges during mechanical ventilation: A) Setting up and connecting a ventilator (Leoni plus, Löwenstein Medical, Bad Ems, Germany) with a humidification system (Fisher & Paykel Healthcare Limited, Auckland, New Zealand). The time taken for this task was recorded. B) Initiating a ventilator and configuring ventilation parameters and alarm limits for a postoperative patient (newborn, 3 kg body weight, with healthy lungs). C) Adjusting mechanical ventilation based on a blood gas analysis indicating respiratory acidosis or alkalosis. D) Modifying ventilator settings in response to sudden improvements or deteriorations in lung compliance, as indicated by corresponding device alarms. The first two tasks were consistent across both versions of PST. Following each intervention, the participants were re-tested using the remaining task and the previously unassigned TT and PST.

We agreed on MV treatment goals consisting of a lung-protective ventilation strategy for all patients using synchronized mandatory intermittent ventilation (SIMV) and HFOV as a rescue MV mode. We specified recommendations regarding the initiation of MV, ventilator setup, and settings for different weight categories from 0.5 to 80 kg. The specifications referred to PIP, PEEP, inspiration time, respiratory rate, pressure support, trigger sensitivity, and TV and minute volume for SIMV; and mean airway pressure, amplitude, frequency for HFOV. In addition, we defined oxygenation targets to avoid hyperoxia, MV monitoring frequency, alarm limit ranges for all respirator settings, and the use of humidified gas for patients ventilated longer than 24 h [6, 7, 23]. We emphasized a standardized display setting showing pressure, flow, volume curves, and alarm limits.

Before and after the intervention, we performed random and unannounced checks of the ventilator and monitor settings in all invasively and non-invasively ventilated patients in both ICUs. The checks were performed approximately twice per week, but not on consecutive or fixed days, and at varying day times to ensure unpredictability (Fig. 1). In addition, ventilator displays and alarm limits were checked. Treatment goal violations were categorized as follows: a) ventilator setup: humidification not turned on, or no water in the humidification system; b) ventilator display: absent or incomplete displays of pressure, volume, flow curves or alarm limits; c) volume target monitoring: missing or non-optimal (inappropriately high or low) limits for minute volume or TV; d) pressure target monitoring: delta pressure > 15 mmHg, PIP > 30 mmHg; missing, or inappropriately high or low limits for PEEP or CPAP pressure; e) saturation limits (SpO2) monitoring: inappropriately high or low limits. The evaluations were conducted discreetly and the staff was not provided with specific details or parameters that were being evaluated.

### Statistical analysis

A required minimum sample size of *N* = 44 participants was calculated to detect a 10% improvement in participants’ overall performance, assuming an overall performance mean of 75% and a standard deviation of 15% with a specified power of 90% and a significance level of 0.05. Continuous variables were expressed as mean (95% confidence interval [CI]). A random allocation sequence was generated using the sample function in R with a 50% probability. Discrete data were compared between groups with the Chi-square test, and effects were reported as Cramer’s V effect sizes. A paired two-tailed t-test was used to compare continuous variables before and after the intervention, and effects were reported as Cohen’s D effect sizes. For the performance comparison, only those participants who had taken part at both test times were included. Linear regression models were calculated for predictor variables (study phase, profession, professional experience) to analyze their impact on the participants’ theoretical knowledge and practical skills performance. Treatment goal adherence over the study period was visualized using a statistical process control chart and adherence rate analyzed using a mixed-effects model to account for multiple checks per patient. *P* values less than 0.05 were considered significant. Statistical analyses were performed using R 4.1.2 (2021–11-01) (R Core Team, Vienna, Austria).

## Results

In this study, a total of 47 nurses and 20 physicians were evaluated both before and after the introduction of the initiative, utilizing the TT. Additionally, 34 nurses and 20 physicians underwent assessment using the PST component of the initiative. Among the participants, 26 individuals (comprising 17 nurses and nine physicians) were tested using the TT only once before the initiative was implemented. On the other hand, 23 participants (consisting of 17 nurses and six physicians) were tested using the TT only once after the initiative was introduced. For the PST component, 39 participants (comprising 30 nurses and nine physicians) were assessed only before the intervention took place. Subsequently, nine participants (including seven nurses and two physicians) were evaluated using the PST component after the implementation of the initiative. At the time of the research, we had a total of 45 nurses working in our NICU and 75 nurses in our PICU. Regarding the medical team, most physicians were permanently assigned to one ward, with 18 physicians in the NICU and 12 in the PICU. However, some team members did have occasional assignments in both areas.

The reasons for taking a test only once were: dismissal, maternity leave, or unavailability. Supplementary Fig. 1 shows the recruitment of participants for the TT and PST and the analyses performed. To compare participants’ performance, we included only those who had completed the tests at both time points (Table 1).

**Table 1:** Characteristics of participants who were tested before and after the intervention

	**Nurses**	**Physicians**
**Theory Test**		
Number of participants	*n* = 47	*n* = 20
Professional experience, years		
0–5	17 (36.2)	15 (75)
6–15	11 (23.4)	3 (15)
> 15	19 (40.4)	2 (10)
**Practical Skill Test**		
Number of participants	*n* = 34	*n* = 20
Professional experience, years		
0–5	12 (35.3)	15 (75)
6–15	10 (29.4)	3 (15)
> 15	12 (35.3)	2 (10)

^*^Categorial variables are shown as counts (percentage)

**Fig. 2:**
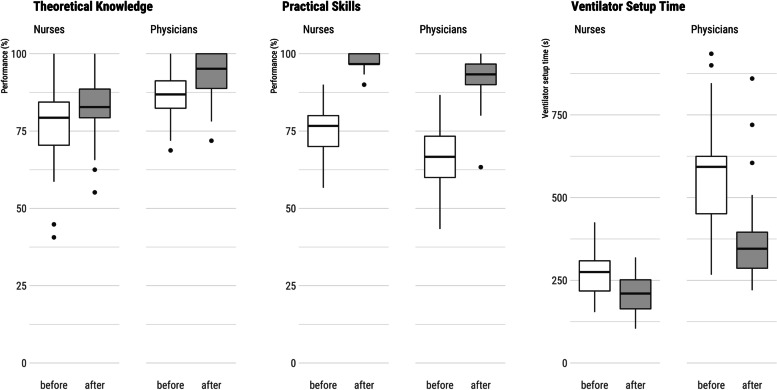
Theoretical knowledge, practical skills, and time for ventilator setup of nurses and physicians pre- and post-intervention

Staff TT and PST performance in percent (CI) improved significantly after the intervention compared to before (TT: 86 [83.1–88.0]% vs. 80 [77.2–82.9]%,* P* < 0.001, d = 0.61; PST: 95 [93.8–97.1]% vs. 73 [69.7–75.5]% vs., *P* < 0.001, d = 2.2). A subgroup analysis by professional groups also showed a substantial improvement in TT performance among nurses (before: 77.4 [73.9–80.9]%, after: 82.6 [79.9–85.3]%, *p* < 0.001, d = 0.562) and physicians (86.2 [82.3–90.1] vs. 92.6 [88.8–96.4]%, *P* = 0.005, d = 0.699). Also, both professions performed better in the PST after the intervention (nurses before: 75.9 [72.9–78.9]% vs. after: 97.5 [96.6–98.5]%, *p* < 0.001, d = 2.35; physicians before: 67 [61.6–72.4]% vs. 92 [88.2–95.8]%, *P* < 0.001, d = 2.11). Physicians (before: 578 [495–661] s vs. after: 390 [313–468] s, *P* < 0.001, d = -0.90), and nurses (before: 273 [249–297] s vs. after: 208 [189–227] s, *P* < 0.001, d = -0.92) needed significantly less time (mean [CI]) for ventilator setup after the intervention than before (Fig. 2). Participants performed similarly in the two TTs before the intervention (TT1 78.3 ± 11.6 vs, TT2 76.9 ± 9.8, *p* = 0.222), indicating the comparability of the two test versions.

**Fig. 3:**
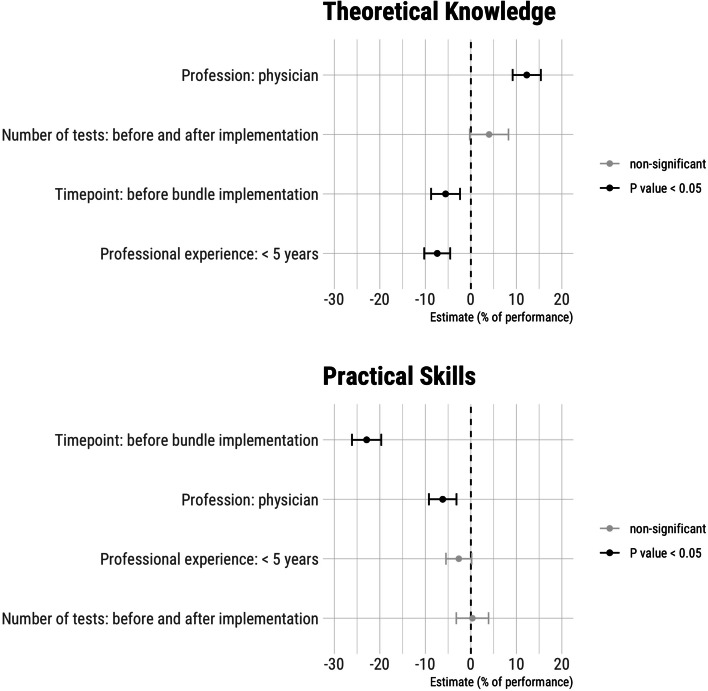
Predictors of theoretical knowledge and practical skills performance

To identify the predictors of staff performance, we analyzed the intervention itself, the participants’ professional experience, the professional group, and the testing regime on the participants’ TT and PST performance by computing linear regression models. The study phase (timepoint) had the most substantial impact on staff performance in percent (CI) (TT: -5.6 [-8.7–2.4]%, *P* < 0.001; PST: -22.9 [-26.1–-19.7]%, *P* < 0.001). Physicians performed better than nurses in the TT (12.3 [9.17–15.4]%), but worse in the PST (-6.2 [-9.2– -3.2]%). Inexperienced staff (professional experience < 5 years) performed worse in the TT than more experienced staff (-7.38 [-10.2– -4.5]%,* P* = 0.001), whereas this effect was not true for the PST (-2.6 [-5.47–0.17]%, *P* = 0.07). The testing regime (number of tests absolved) had no impact on performance, indicating that whether a candidate was tested before and after or only once after the intervention was irrelevant (1.8 [-1.6–5.3]%, *P* = 0.30) (Fig. 3).

Self-confidence among both professions increased significantly after bundle implementation (Nurses: *p* = 0.048; Physicians: *p* = 0.02) as Supplementary Figure 2 illustrates. Supplementary Figure 3 shows the connection between work experience, occupational group, and performance. Practical Skill Test performance improvement appeared to be largely independent of staff experience (mean improvement: 0–5 years: 24%, 6–15 years: 22%, > 15 years: 20%). However, analyzing the theory test results, we noticed that inexperienced staff showed substantial improvements (0–5 years: 5%), as did the highly experienced ones (> 15 years: 5%). Meanwhile, employees with a moderate level of experience demonstrated improvements in individual topics but did not show a relevant overall mean theory test performance increase (6–15 years: 1%). The theory test revealed certain topics that posed significant challenges for the participants, namely Intrapulmonary shunts, Ventilator-associated pneumonia, and High-frequency oscillatory ventilation. As for the practical skills test, participants, including both nurses and physicians, struggled the most with alarm management, display setting, inspiratory time choice, and ventilation adjustments for compliance changes.

**Fig. 4:**
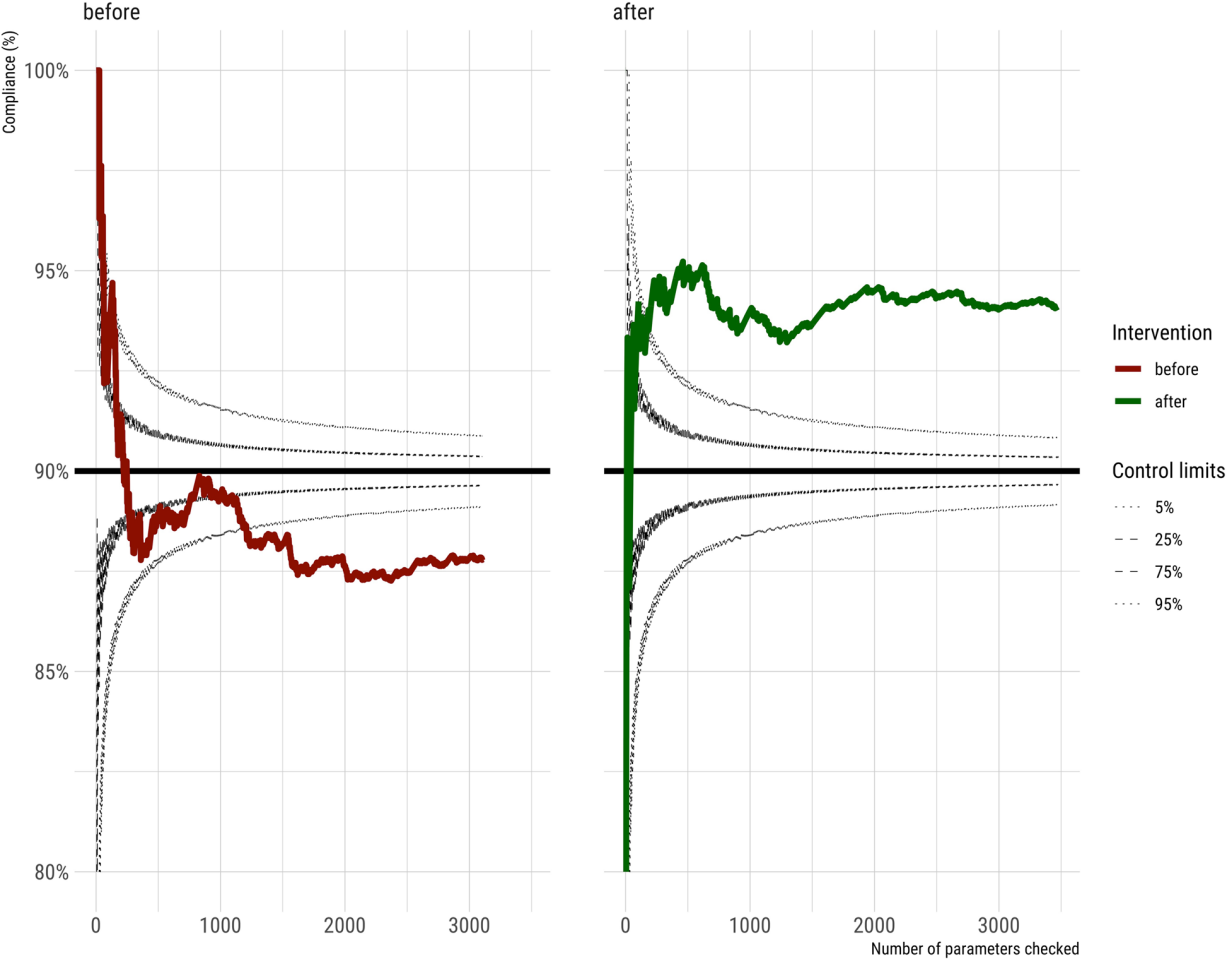
Statistical process control chart illustrating adherence to treatment goals (black horizontal line) with control limits before (dark red) and after (dark green) the implementation of an intervention bundle to improve the quality of mechanical ventilation in children plotted as running means. The checks covered ventilator setup, ventilator display, volume, pressure, and pulse oximetry target monitoring

A total of 3103 parameters were examined during 322 random checks in 105 MV patients pre-intervention (October 2019–June 2020) and a total of 3476 parameters were evaluated during 340 random checks in 108 MV patients post-intervention (January 2021–July 2021) (Fig. 1). The post-intervention compliance with treatment goals was significantly and persistently above the target of 90% (Fig. 4).

A mixed-effects model with a random term (Patient ID) that accounted for the multiple checks per patient confirmed that the mean compliance increased significantly by 6.7% from 87.8% before the intervention to 94.5% (*P* < 0.001) (see also Supplementary Table 1), with slightly lower compliance in the PICU than in the NICU.

## Discussion

Implementing a pediatric MV educational program improved theoretical knowledge and practical skills in both nurses and physicians, and increased compliance with treatment goals in two pediatric intensive care units.

We report significant post-intervention improvements in theoretical knowledge and practical skill performance among both professional groups. However, staff had already performed strongly during baseline evaluation (Fig. 2). Nurses scored lower on the TT compared to physicians. In contrast, nurses performed better than physicians in the PST and ventilator setup. We expected this result due to the local task assignments. The implementation significantly improved physicians’ practical skills and significantly reduced the time that physicians and nurses needed to set up a ventilator. The staff of both professions with limited experience (0–5 years) scored lower in the TT than more experienced staff (> 5 years). Inexperience was not a relevant factor in PST performance (Fig. 3), indicating that nurses, in particular, acquire theoretical knowledge with increasing professional experience. The notable drop-out rates within our study, particularly evident in the TT group (nurses: 26%, physicians: 10%) and even more prominently in the PST group (nurses: 47%, physicians: 24%), can be attributed to a combination of factors. These factors encompass staff turnover, the intricate nature of the testing environment which took place during regular working hours, and the anxiety-inducing testing scenario that deterred certain participants from undergoing the test a second time.

Supplementary Figure 3 illustrates the correlation between staff experience and performance in theory and practical skill tests. Surprisingly, we observed that practical skill test performance improvement was largely independent of staff experience (mean improvement: 22%). However, for the theory test, there was an interesting trend: inexperienced and highly experienced staff showed significant improvements, while moderately experienced staff demonstrated improvements in individual topics but no significant overall increase in performance. In the theory test, Intrapulmonary shunts, Ventilator-associated pneumonia, and High-frequency oscillatory ventilation were identified as particularly challenging topics for the participants. On the other hand, the practical skills test revealed that both nurses and physicians faced difficulties with alarm management, display settings, inspiratory time selection, and making ventilation adjustments for compliance changes.

These results suggest that training initiatives should cater to both inexperienced staff and highly experienced individuals. In our setting, physicians scored lower than nurses in practical skills, indicating a potential lack of exposure to practical aspects, possibly influenced by task distribution within our clinic. Tailored training to address specific knowledge gaps would be highly beneficial.

We used an educational film (www.uke.de/picu-nicu) available to the team as a self-learning offering which was well adopted (Fig. 1) and proved to be a resource-effective and efficient measure for standardized and contemporary training [24]. We tested the theoretical knowledge of the participants but moreover their practical skills in using the equipment and their abilities to respond to clinical challenges using OSCE. Although OSCE can only partially reflect clinical reality [14, 18, 21], in our study setting, OSCE was well suited to test the psychomotor skills of the participants.

Our aim was to establish a standardized and inclusive educational approach that would enhance the understanding and awareness of the tasks and challenges faced by both professional groups. This objective aligns with the fundamental principles of effective interprofessional education, as outlined by van Diggele [17]. By promoting interprofessional education, we sought to diminish hierarchical barriers and empower nursing professionals to actively engage and contribute their expertise, ultimately enhancing patient safety. The notable increase in self-confidence observed among the participants of our study suggests that both professions have gained a heightened level of proficiency in the field of mechanical ventilation in children (Supplementary Fig. 2). When designing our educational program, we carefully considered the guiding principles put forth by van Diggele et al., which delineate key considerations for planning and implementing interprofessional facilitation in both classroom and clinical settings [17].

With patient safety, the high staff turn-over [25], and the high proportion of inexperienced staff in intensive care units in mind, a practical, consistent, and resource-effective education is of great importance in health care [19, 20].

This initiative defined numerous aspects regarding ventilator setup, the visible display of all relevant parameters, and specifications for alarm limits and patient monitoring during MV. Compliance with these treatment goals was only accessible through random checks of patients and respirators. The mean compliance to treatment goals rose rapidly by about 7% after the intervention, well above the aim of 90%, and remained at this high level (Fig. 4). This effect was also significant after correcting for multiple checks in some patients calculating a mixed-effects model (sTable 1). Incorrect respirator setup, settings, and missing alarm limits occurred significantly less frequently after the intervention, reflecting higher team compliance with treatment goals. To our knowledge, this study is the first to report improvements in the team’s theoretical knowledge, practical skills, and immediate improvements in compliance with MV treatment goals in actual pediatric patients after an educational intervention.

Future studies should investigate whether improved team knowledge and optimized ventilator settings result in better patient outcomes and enhanced patient safety for ventilated children. This may require validating the instruments used in this study and establishing a collaborative multi-center, regional, or national training program in partnership with professional organizations.

### Limitations

We conducted the study at only one institution. Our approach involved a comprehensive bundle of measures, making it challenging to isolate the specific impact of each component retrospectively. The distribution of roles in the setup of ventilators and their adjustment may vary locally, and additional specialized professionals may be involved in respiratory management, thus restricting the generalizability of the results. In addition, pediatric ventilator management is a complex skill, and neither international recommendations nor universal agreements exist among experts regarding MV goals for children. We, therefore, defined local treatment goals and MV settings and adapted the educational program to local requirements. Although we did not determine the difficulty level of the two TTs and PSTs, the cross-over testing study design, coupled with randomized assignment and per-participant analysis, minimized the potential impact of any variations, thus providing a robust evaluation of the intervention’s effectiveness. Participants performed similarly in the two TTs and PSTs before the intervention, that a similar level of difficulty of the tests can be assumed. Blinding of the TT and PST assessors and random checks were impossible because of the study design. Knowing that they participated in the initiative, the treating team may have changed their behavior (Hawthorne effect).

## Conclusions

Implementation of a pediatric mechanical ventilation education bundle significantly improved theoretical knowledge, practical skills, and self-confidence among interprofessional intensive care staff and increased treatment goal compliance in actual pediatric patients of two intensive care units.

### Supplementary Information

**Additional file 1. **Checklists for ventilator setup and initial settings, educational materials, theory tests (TT), and practical skill tests (PST).

**Additional file 2: Supplementary Figure 1.** The flowchart shows the number of pre-and post-intervention recruited participants and the statistical analyses performed.

**Additional file 3: Supplementary Figure 2.** Self-confidence of nurses (top) and physicians (bottom) before vs. after a pediatric mechanical ventilation education intervention.

**Additional file 4: Supplementary Figure 3.** A) Average performance of participants in the Theory Test Category and the Practical Skill Test Topic Category, categorized by their respective professional groups and experience. B) Detailed view of the performance related to each individual aspect of the practical skill test, as well as for the individual topics of the theory test, separately for the two occupational groups, nurses, and physicians. The dashed vertical red lines represent the average performance before the intervention, while the solid lines depict the performance after the intervention. The gray diamonds indicate the mean performance per topic or task of the tests before the intervention, and the black diamonds represent the mean performance after the intervention. iNO: Inhaled Nitric Oxide, HFOV: High Frequency Oscillatory Ventilation, DOPES: Acronym: Dislocation, Obstruction, Pneumothorax, Equipment, Stomach, FiO2: Fraction of Inspiratory Oxygen.

**Additional file 5: sTable 1.** Mixed-effects model for treatment goal compliance.

## Data Availability

The datasets used and/or analysed during the current study are available from the corresponding author on reasonable request.

## Abbreviations

HFOV: High-frequency oscillatory ventilation
ICU: Intensive Care Unit
IPE: Interprofessional Education
NICU: Neonatal Intensive Care Unit
PEEP: Positive end-expiratory pressure
PICU: Pediatric Intensive Care Unit
PIP: Peak inspiratory pressure
PST: Practical Skill Test
MAP: Mean arterial pressure
MV: Mechanical Ventilation
SIMV: Synchronized mandatory intermittent ventilation
TV: Tidal volume
TT: Theory Test
OSCE: Objective Structured Clinical Examination
