# Supplementary material for: Pilot study of an interprofessional pediatric mechanical ventilation educational initiative in two intensive care units
Source: BMC Med Educ. 2023 Aug 28;23:610. doi: 10.1186/s12909-023-04599-1 (PMC10463469; doi:10.1186/s12909-023-04599-1)
Supplement: Supplementary file 1 — Additional file 1. Checklists for ventilator setup and initial settings, educational materials, theory tests (TT), and practical skill tests (PST). [file 12909_2023_4599_MOESM1_ESM.pdf]

KiNDER UKE

Universitätsklinikum Hamburg-Eppendorf UKE HAMBURG

Section Neonatology and Paediatric Intensive Care Medicine | Sabine Dreger, Thormen Höfeler, Philipp Deindl

Invasive ventilation in infants and children

Problems:

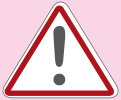

D ... Dislocation (tube position)

O ... Obstruction (of the tube or airway)

P ... Pneumothorax

E ... Equipment (ventilator, tubes)

S ... Stomach (distension)

S ... Synchrony with the ventilator/ sedation

Control of ventilation:

Ventilation problems:

- Hypocapnia: Reduce respiratory minute volume (reduce frequency, reduce driving pressure*)
- Hypercapnia: Increase respiratory minute volume (increase frequency, increase driving pressure*)

Oxygenation problems:

Hypoxia (SpO_2 ↓)/Hypoxaemia (paO_2 ↓): Increase FiO_2, increase PEEP

Abbreviations:

***Driving Pressure:** Difference PIP – PEEP | **FiO2:** Fraction of inspired oxygen | **Freq:** Frequency | **MAP:** Mean airway pressure | **(R)MV:** (Respiratory) Minute volume | **paO2:** partial pressure of arterial oxygen | **PEEP:** Positive end-expiratory pressure | **PIP:** Peak Inflation Pressure | **SIMV:** Synchronized Intermittent Mandatory Ventilation | **SpO2:** Pulse oxymetric oxygen saturation |

Section Neonatology and Paediatric Intensive Care Medicine | Sabine Dreger, Thormen Höfeler, Philipp Deindl

Failure of conventional SIMV ventilation

NO ventilation:

- Begin with 20 ppm
- Ideally, echocardiography beforehand
- *CAVE*: Heart valve defects with dependent right-left shunt

Risks:

- Methaemoglobinaemia (Met-Hb max. 2.5 %)
- Nitrogen dioxide formation (max. 0.5 ppm)

HFO-Ventilation:

Start settings:

- MAP 2 points above previous P_{mean} (e.g. 10-12 cm H_2O)
- Amplitude (Δp 10-30 cm H_2O)
- Frequency ((8)-10-(12) Hz)

→ ABG and X-ray check promptly, watch for thoracic excursion

Control:

- Hypercapnia: Reduce Freq. (Hz), increase amplitude
- Hypocapnia: Increase Freq. (Hz), reduce amplitude
- Hypoxia: Increase MAP and FiO_2 erhöhen

Abbreviations:
FiO2: Fraction of inspired oxygen | **Freq:** Frequency | **MAP:** Mean airway pressure |

Section Neonatology and Paediatric Intensive Care Medicine | Invasive ventilation in infants and children

SIMV ventilation – Useful Start-up settings

CAVE: These values are only guidelines, always adapted to the clinical situation and experience of the user.

Parameters	0,5 kg	1 kg	2 kg	3 kg	4 kg	5 kg
PIP (cmH_2O)	15	15	15	15	15	15
PEEP (cmH_2O)	5	5	5	5	5	5
Flow (l/min)	8	8	8	10	12	12
Frequency (/min)	65	60	55	45	40	35
T insp (sec)	0,30	0,33	0,35	0,38	0,40	0,45

Trigger volume 15%, VTG/VTLim off

Parameters	10 kg	15 kg	20 kg	30 kg	40 kg	50 kg	60 kg
PIP (cmH_2O)	15	15	15	15	15	15	15
PEEP (cmH_2O)	5	5	5	5	5	5	5
Frequency (/min)	20	25	20	18	15	15	15
T insp (sec)	0,6	0,7	0,8	1,0	1,1	1,1	1,1
Rampe (sec)	0,2	0,2	0,2	0,2	0,2	0,2	0,2

Abbreviations: **T insp:** Inspiration time

SIMV ventilation – Useful Start-up settings

- Set FiO_2 according to paO_2/peripheral saturation

→ CAVE for heart valve defects

→ SpO_2 upper limit for FiO_2 >0,21: 98% (newborns 97%, premature babies 95%)

- I:E ratio ideally 1:2, ensure sufficient expiration (CAVE: Auto-PEEP (termination of expiration in volume/ flow curve))
- Target VT 4-6 ml/kg body weight
- Moist ventilation for all newborns/infants or for prolonged ventilation time
- Filter ventilation with planned ventilation time <24 hours

Alarm limits:

VT high: 8 ml/kg

Peak pressure: 5 cm H_2O over PIP

VT low: 3 ml/kg

Frequency: default Freq. + 10

Respiratory minute volume based on age and weight (newborns approx. 200 ml/kg, adults 100 ml/kg)

Abbreviations: **FiO2:** Fraction of inspired oxygen | **Freq:** Frequency | **SpO2:** Pulse oxymetric oxygen saturation | **VT:** Tidal volume

Supplementary Material

Pilot Study of an Interprofessional Pediatric Mechanical Ventilation Educational Initiative in Two Intensive Care Units

Theory Test 1

Pseudonym/ID: ________________

Professional group

O Nurse O Physician

Work experience

O 0 to 5 yrs O 6 to 10 yrs O 11 to 15 yrs O 16 to 20 yrs O +20 yrs

Please rate your level of confidence in using ventilation in children.

O very confident O confident O unconfident O very unconfident

Please indicate the correct statements by checking the corresponding boxes (multiple answers are possible).

---

1. What is/are the *desired* effect(s) of inhaled nitric oxide (iNO)?
    a. Improvement of lung perfusion
    b. Secretolysis
    c. Reduction of pulmonary resistance
    d. Increase in pulmonary resistance

2. What is/are the adverse side effect(s) of inhaled nitric oxide (iNO)?
    a. Hepatotoxicity
    b. Met-Hb formation
    c. Anuria

3. Please indicate which value(s) increase(s) with a ventilation disorder of the lungs:
    a. pO_2
    b. pCO_2
    c. Sodium
    d. SaO_2

4. Please select the change(s) in ventilation parameters that improve CO_2 elimination:
    a. Increasing the ventilation frequency
    b. Increase of the peek inspiratory pressure (PIP)
    c. Increase of FiO_2
    d. Reduction of the ventilation frequency

5. Please indicate which change(s) in ventilation settings can improve oxygenation:
    a. Increase FiO_2
    b. Increase PEEP
    c. Reduce PiP
    d. Increase ventilation frequency

6. Please indicate the possible causes of the patient's acute deterioration under mechanical ventilation:
    a. Tube dislocation/ misalignment
    b. Tube obstruction/ displacement
    c. Tension pneumothorax
    d. Highly distended gastrointestinal tract

7. Please select the correct definition(s) of "tidal volume":
    a. The volume administered by ventilation per minute
    b. The volume administered per breath
    c. The volume that remains in the lungs after expiration
8. What is the recommended target range for tidal volume in lung-protective ventilation?
    a. 1-2 ml/kg
    b. 4-8 ml/kg
    c. 10-12 ml/kg
    d. 15-20 ml/kg
9. Please select the effect(s) of an *increase* in positive end-expiratory pressure (PEEP):
    a. Improvement of oxygenation
    b. Opening of collapsed lung areas
    c. Keeping the airways and alveoli open
    d. Improvement of venous return to the heart
10. Please mark the correct statements about intrapulmonary right-to-left shunts by checking the appropriate box(es):
    a. It is a congenital heart defect
    b. It is caused by the fact that unventilated areas of the lungs are not well supplied with blood
    c. Intrapulmonary shunts can worsen blood oxygenation
11. Please indicate the correct risk factors for ventilator-associated pneumonia (VAP)?
    a. Microaspirations of secretion
    b. Ventilation longer than 72 hours
    c. Positioning the patient flat (head end all the way down)
    d. No oral hygiene

12. Please select the correct definition of pressure amplitude during high-frequency ventilation (HFOV):

    a. It is the difference between P_{max} and P_{min}

    b. It is another description for "overpressure valve"

    c. It is the time needed to build up the PIP

13. Please indicate which change(s) in ventilation settings for high-frequency oscillatory ventilation (HFOV) can *improve* oxygenation:

    a. Increase in MAP

    b. Increase in FiO_2

    c. Increase of HFOV-frequency (Hz)

14. Please indicate which change(s) in ventilation settings for high-frequency oscillatory ventilation (HFOV) can *reduce* CO_2 in the blood:

    a. Reducing HFOV-frequency (Hz)

    b. Increasing the amplitude

    c. Increase of FiO_2

    d. Increasing HFOV-frequency (Hz)

15. Please indicate the recommended range for setting the frequency (Hz) in high-frequency oscillatory ventilation (HFOV):

    a. 5-15 Hz

    b. The higher the better

    c. 20-30 Hz

    d. Never higher than the PEEP

Supplementary Material

Pilot Study of an Interprofessional Pediatric Mechanical Ventilation Educational Initiative in Two Intensive Care Units

Theory Test 2

Pseudonym/ID: ______________

Professional group

O Nurse O Physician

Work experience

O 0 to 5 yrs O 6 to 10 yrs O 11 to 15 yrs O 16 to 20 yrs O +20 yrs

Please rate your level of confidence in using ventilation in children:

O very confident O confident O unconfident O very unconfident

Please indicate the correct statements by checking the corresponding boxes (multiple answers are possible).

---

1. What results in the respiratory minute volume?

    a. Tidal volume (TV) x Respiratory rate

    b. Tidal volume (TV) = Bodyweight/4+4

    c. It is the number of breaths per minute

    d. Age x Respiratory rate

2. What is/are the effect(s) of positive end-expiratory pressure (PEEP) during ventilation?
    a. Keeping the airways and alveoli open
    b. Improvement of oxygenation
    c. Maintenance of blood pressure
    d. Improvement of diuresis
3. What can be the cause(s) for the alarm: "respiratory minute volume too low" on the ventilator?
    a. Tube obstruction/ displacement
    b. Tube dislocation/ misalignment
    c. Device error
    d. Very stiff lungs
4. What can lead to a rise in CO_2 under ventilation?
    a. Pneumothorax
    b. Deeper sedation with less self-breathing of the patient
    c. Reopening of an atelectasis
    d. High FiO_2 set
5. What tidal volume (in ml/kg) should a patient receive in terms of a lung-protective mechanical ventilation scheme?
    a. 1-2
    b. 4-8
    c. 10-12
    d. 15-20

6. Which parameter(s) can improve Oxygenation?

   a. Increase of FiO_2

   b. Increase of the inspiration time (Ti)

   c. Increase of the positive end-expiratory pressure (PEEP)

   d. Decrease of the peak inspiratory pressure (PIP)

7. What change(s) in ventilation settings can one use in high-frequency oscillatory ventilation (HFOV) to lower the CO_2 in the blood during ventilation?

   a. Increase amplitude

   b. Lower frequency (Hz)

   c. Increase frequency (Hz)

   d. Increase FiO_2

8. What is/are possible indication(s) for a high-frequency oscillatory ventilation (HFOV)?

   a. Pronounced oxygenation disorder

   b. Pronounced hypercapnia

   c. Severe lung failure (ARDS)

   d. HFOV is a common alternative to CPAP

9. What is/are the effect(s) of inhaled nitric oxide (iNO)?

   a. Reduction of pulmonary resistance

   b. Improvement of the lung blood circulation

   c. Lowering the skin temperature

   d. Antibacterial effect

10. What are strategies to avoid a ventilator-associated pneumonia (VAP)?

    a. 30° upper body elevation

    b. Regular oral hygiene

    c. Regular and sterile suction

    d. Bonding

11. You don't see chest excursions on the safely intubated patient. What could be the cause(s)?

    a. Pneumothorax

    b. Tube obstruction/displacement

    c. Thoracic rigidity/stiffness

    d. Asynchrony (Ventilator and patient are not "in sync")

12. What observation(s) suggest poor ventilation of the lungs?

    a. Increase of pCO_2

    b. Decrease of SpO_2

    c. Alarm on the ventilator: "respiratory minute volume too high"

    d. Oxygen concentration in the blood = paO2 >100 mmHg

13. What is meant by "dead space" in the context of ventilation?

    a. Room in which nothing happens

    b. Space of the respiratory system that is *not* involved in pulmonary gas exchange (e.g., tube system)

    c. Space that remains in expiration

14. What is/are adverse side effect(s) of inhaled nitric oxide (iNO)?

    a. Hepatotoxicity

    b. Met-Hb formation

    c. Anuria

15. In which range should the frequency (Hz) in high-frequency oscillatory ventilation (HFOV) be set for ventilation?

    a. 5-15 Hz

    b. The higher the better

    c. 20-30 Hz

    d. Never higher than the PEEP

Practical Skill Test 1

Pseudonym/ID

**Task 1: Leoni Ventilator (Heinen und Löwenstein) Set up**		
	Ventilator plugged into the socket	❑
	Grounding plugged in	❑
	Gas connections correctly plugged into the wall connections	❑
	Expiratory valve correctly mounted	❑
	Inspiratory valve correctly mounted	❑
	Expiration hose correctly fitted	❑
	Test lung connected	❑
	Built-in flow sensor	❑
	Flow sensor cable connected	❑
	Pressure measuring line mounted	❑
	Water container punctured	❑
	Humidification system mounted in the socket	❑
	Temperature measuring cable correctly mounted	❑
	**Points Task 1**	/13
	**Time measured**	

**Task 2: Base settings for Leoni Ventilator (Heinen und Löwenstein) 3kg (newborn)**		
**Settings**	Flow sensor calibrated	❑
	Pip: 12-16 cmH2O	❑
	PEEP: 4-6 cmH2O	❑
	Flow: 8-12 cmH2O	❑
	Respiratory rate: 35-45/Min	❑
	Ti: 0.33 - 0.45	❑
	FiO2: 21-30 %	❑
**Alarm limits**	Respiratory minute volume upper limit: 0.40-0.60 L/Min	❑
	Respiratory minute volume lower limit: 0.60- 1.0 L/Min.	❑
	TV upper limit: 21-30 ml	❑
**Display**	Set permanently visible	❑
	Three curves displayed	❑
	Time interval: 5 - 30 sec.	❑
	**Points Task 2**	/13

**Task 3: Response to Arterial Blood Gas Analysis (respiratory acidosis)**		
	Respiratory rate increased by 5-10 /min	❑
	PIP increased by 1-2 cmH2O	❑

**Task 4: Response to TV alarm high, respiratory minute volume alarm high**		
	PIP reduced by 1-2 cmH2O	❑
	Respiratory rate reduced by 5-10 /min	❑

**Total points**	Task 1	____/13
	Task 2	____/13
	Task 3	____/2
	Task 4	____/2
	**Total**	**____/30**

Practical Skill Test 2

Pseudonym/ID

**Task 1: Leoni Ventilator (Heinen und Löwenstein) Set up**		
	Ventilator plugged into the socket	❑
	Grounding plugged in	❑
	Gas connections correctly plugged into the wall connections	❑
	Expiratory valve correctly mounted	❑
	Inspiratory valve correctly mounted	❑
	Expiration hose correctly fitted	❑
	Test lung connected	❑
	Built-in flow sensor	❑
	Flow sensor cable connected	❑
	Pressure measuring line mounted	❑
	Water container punctured	❑
	Humidification system mounted in the socket	❑
	Temperature measuring cable correctly mounted	❑
	**Points Task 1**	/13
	**Time measured**	

**Task 2: Base settings for Leoni Ventilator (Heinen und Löwenstein) 3kg (newborn)**		
**Settings**	Flow sensor calibrated	❑
	Pip: 12-16 cmH2O	❑
	PEEP: 4-6 cmH2O	❑
	Flow: 8-12 cmH2O	❑
	Respiratory rate: 35-45/Min	❑
	Ti: 0.33 - 0.45	❑
	FiO2: 21-30 %	❑
**Alarm limits**	Respiratory minute volume upper limit: 0.40-0.60 L/Min	❑
	Respiratory minute volume lower limit: 0.60- 1.0 L/Min.	❑
	TV upper limit: 21-30 ml	❑
**Display**	Set permanently visible	❑
	Three curves displayed	❑
	Time interval: 5 - 30 sec.	❑
	**Points Task 2**	/13

**Task 3: Response to Arterial Blood Gas Analysis (respiratory alcalosis)**		
	Respiratory rate reduced to 33-37/Min (alt.: reduced by 5-10/Min)	❑
	PIP reduced by 1-2 cmH2O	❑

**Task 4: Response to TV alarm low, respiratory minute volume alarm low**		
	PIP increased by 1-2 cmH2O	❑
	Respiratory rate increased by 5-10/min	❑

**Total points**	Task 1	____/13
	Task 2	____/13
	Task 3	____/2
	Task 4	____/2
	**Total**	**____/30**

Checklist Setup
Ventilator „Leoni plus“ (Heinen & Löwenstein)

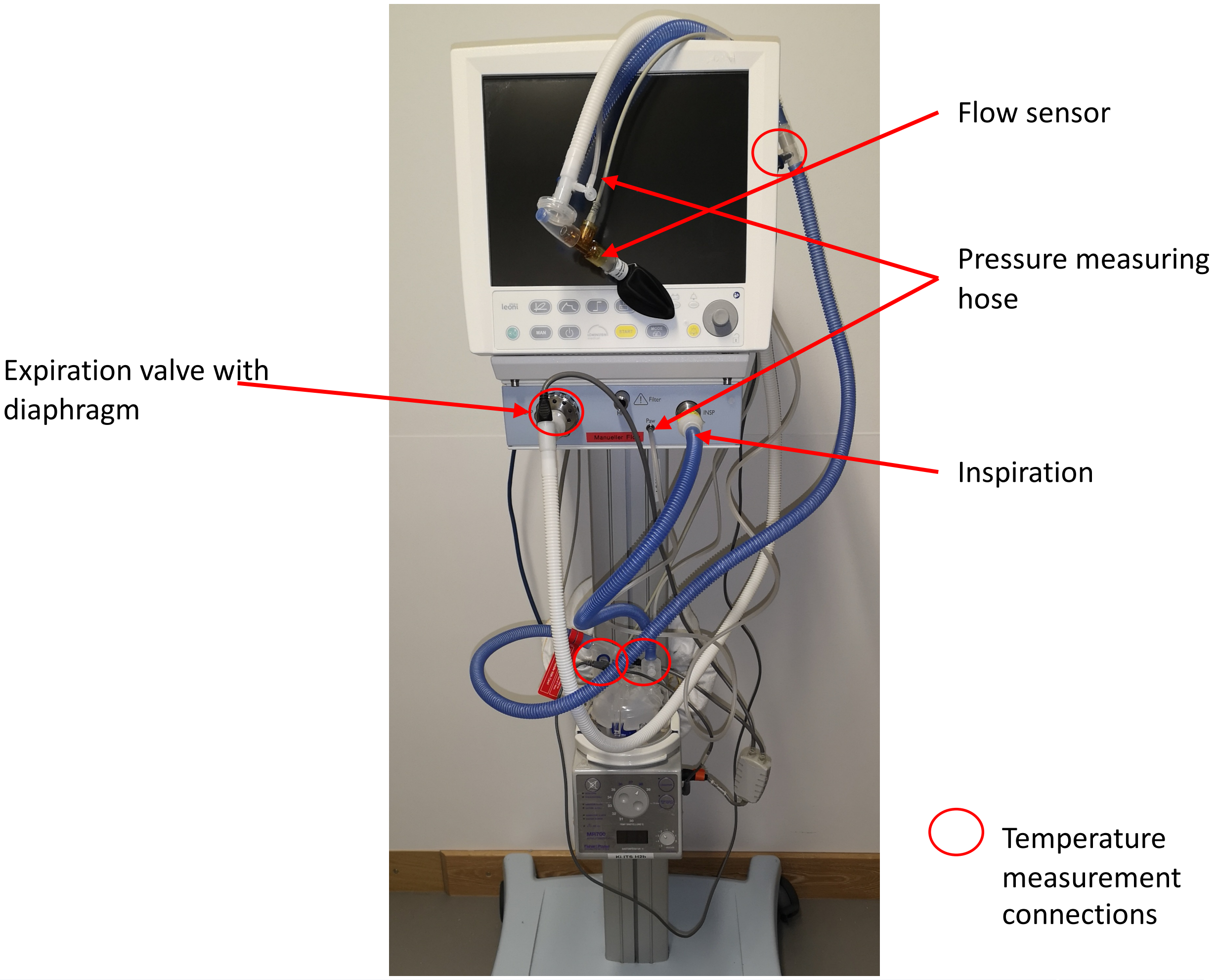

Gas connections (Air and O2) plugged into wall connections	
Power supply ventilator (emergency power) and heater plugged in	
Grounding and data transmission cable (ICM) plugged in	
Expiration valve with diaphragm (lettering "top" legible and wrinkle-free) installed	
Hose system converted (see picture)	
Pressure measuring hose connected	
Flow sensor and test lung connected	
Flowsensor cable plugged in (on flowsensor and back of ventilator)	
Temperature probes (4x) plugged in	
New bottle of sterile aqua dest poked	
Leave remaining adapters in place	

Checklist Setup „Leoni plus“ on HFO

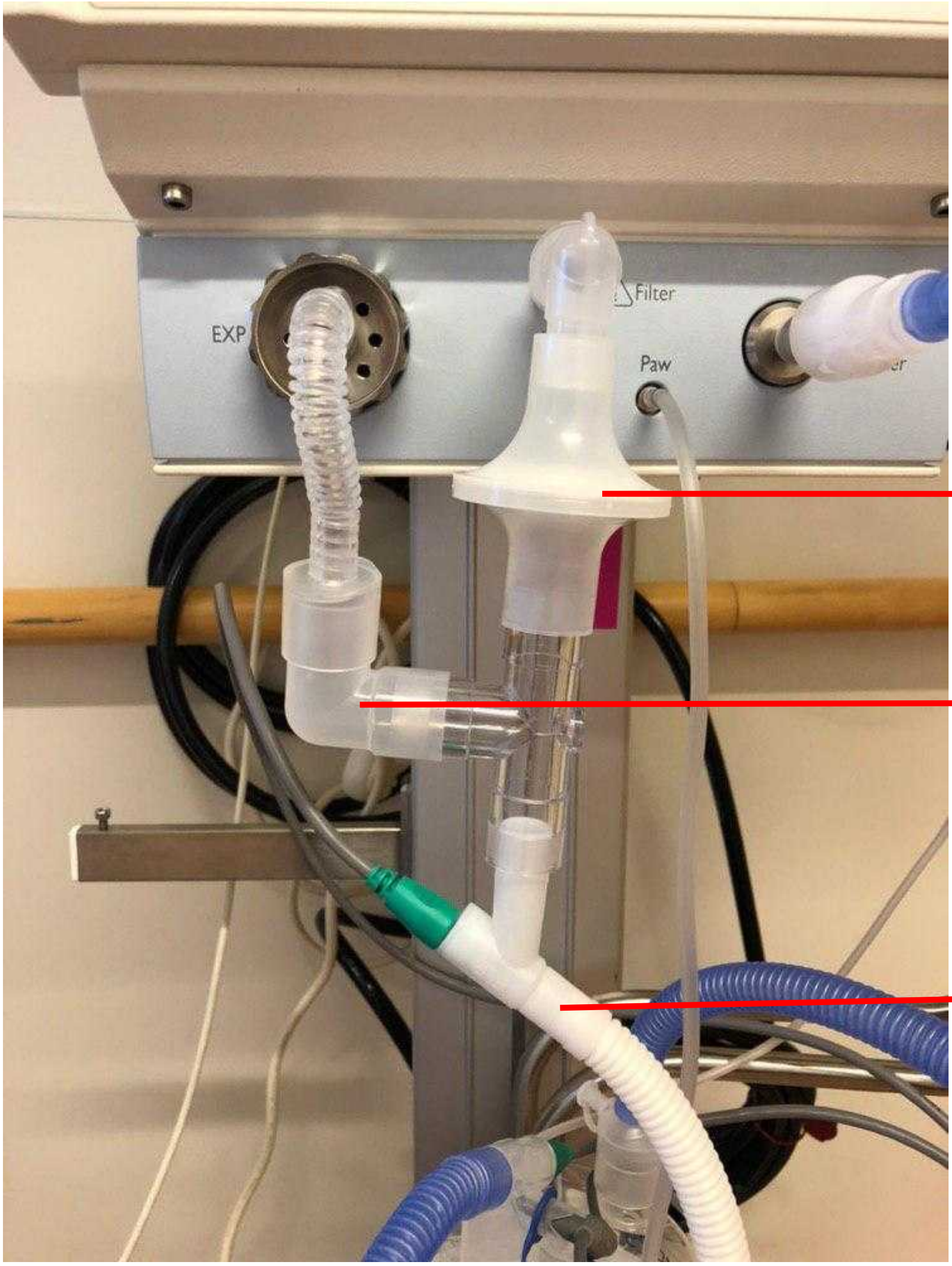

Fit the filter to the middle connection	
Mount connector from expiration valve to filter	
Connect the expiration tube to the filter	

Checklist Setup „Leoni plus“ with NO

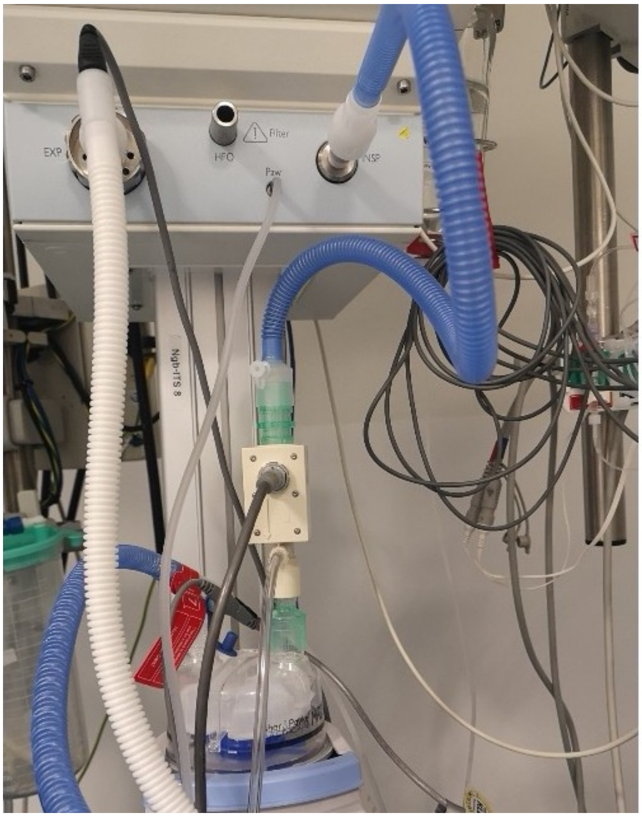

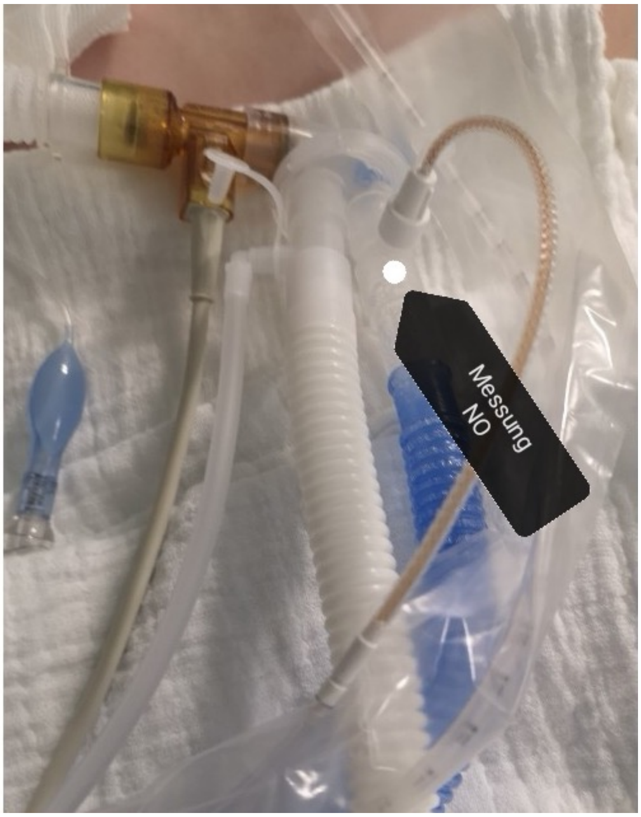

<u>Required adapters:</u><br>Feed-in: connector 22M/15F (close to the device), connector 22F/15M (close to the heating system)<br>NO-measuring: Luerlock RT062	
Install NO feed module with adapter in inspiration flow in front of the heating pot	
Install NO sensor cable for measurement with adapter in inspiratory flow close to patient	

Checklist Start-up
Ventilator „Leoni plus“ (Heinen & Löwenstein) 5H

Switch on ventilator and heating	
System check including expiration valve and leakage testing	
Calibration of the flow sensor	
Selection of the ventilation mode (SIMV)	
Setting the ventilation parameters and alarm limits	
Display of 3 curves (volume, flow, pressure)	
Display measured values page 1: PIP, PEEP, Pmean, MV, VTe, % spontaneous	
Permanent display of the alarm limit values	

**Parameters**	**0,5 kg**	**1 kg**	**2 kg**	**3 kg**	**4 kg**	**5 kg**
PIP (cmH_2O)	15	15	15	15	15	15
PEEP (cmH_2O)	5	5	5	5	5	5
Flow (l/min)	8	8	8	8	10	12
Frequency (/min)	65	60	55	45	40	35
T insp (sec)	0,30	0,33	0,35	0,38	0,4	0,45
MV high (l/min)	*0,15*	*0,30*	*0,60*	*0,90*	*1,20*	*1,50*
MV low (l/min)	*0,08*	*0,20*	*0,30*	*0,45*	*0,60*	*0,75*
VT high (ml)	*4*	*8*	*16*	*24*	*32*	*40*
VT low (ml)	*1,5*	*3*	*6*	*9*	*12*	*15*

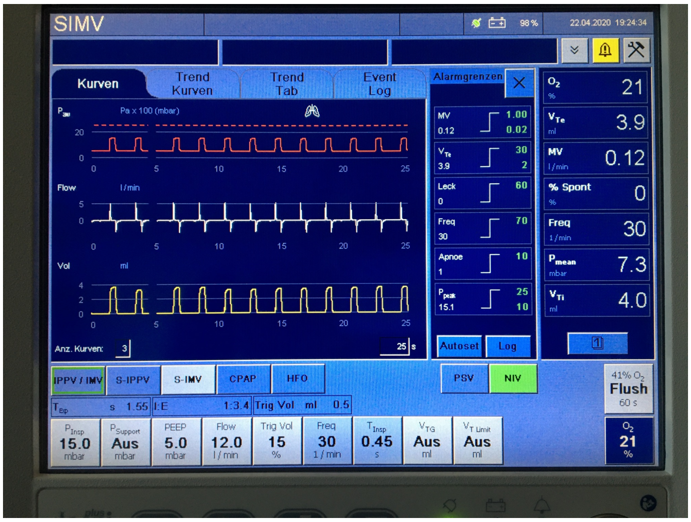

Weight-independent:
Consider pressure support
Trigger volume 15%
VTG/VTLim off

Leak 30%
Apnoea 10 sec
Ppeak 20 cmH_2O
Frequency: Default frequency + 10

Checklist Start-up
Ventilator „Leoni plus“ (Heinen & Löwenstein) K1a

Switch on ventilator and heating	
System check including expiration valve and leakage testing	
Calibration of the flow sensor	
Selection of the ventilation mode (IV → SIMV)	
Setting the ventilation parameters and alarm limits	
Display of 3 curves (volume, flow, pressure)	
Display of measured values p. 1: VT, MV, % spontaneous, FiO2, Pmean, leakage	
Permanent display of the alarm limit values	

Parameters	1 kg	2 kg	3 kg	4 kg	5 kg
PIP (cmH_2O)	15	15	15	15	15
PEEP (cmH_2O)	5	5	5	5	5
Flow (l/min)	8	8	8	10	12
Frequency (/min)	60	55	45	40	35
T insp (sec)	0,33	0,35	0,38	0,4	0,45
MV high (l/min)	*0,30*	*0,60*	*0,90*	*1,20*	*1,50*
MV low (l/min)	*0,15*	*0,30*	*0,45*	*0,60*	*0,75*
VT high (ml)	*8*	*16*	*24*	*32*	*40*
VT low (ml)	*3*	*6*	*9*	*12*	*15*

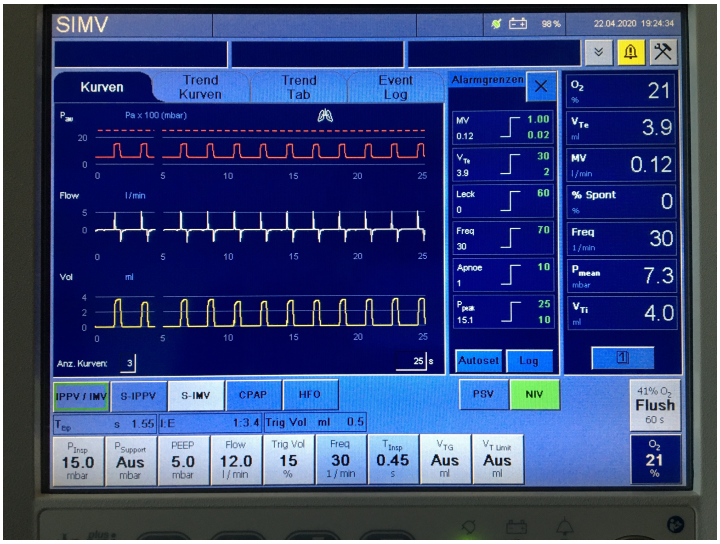

Weight-independent:
Consider pressure support
Trigger volume 15%
VTG/VTLim off

Leak 30%
Apnoea 10 sec
Ppeak 20 cmH_2O
Frequency: Default frequency + 10

Checklist Setup
Ventilator „Evita® 500“ (Dräger)

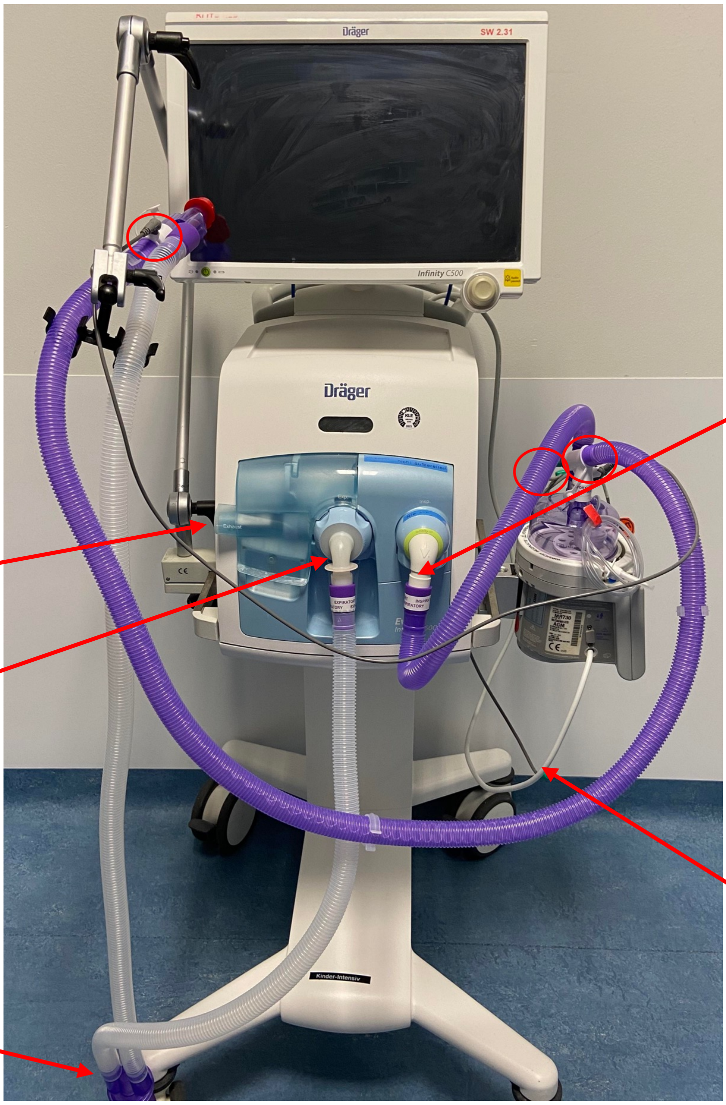

Inspiration block

Flow sensor

Expiration valve with diaphragm

Connections temperature measurement

4th heating cable not required

Water trap at the lowest point

Gas connections (Air and O2) plugged into wall connections	
Power supply ventilator (emergency power) and heater plugged in	
Grounding and data transmission cable (ICM) plugged in	
Expiration valve installed	
Hose system connected	
Flow sensor (lettering above) and test lung connected	
Humid ventilation: temperature probes plugged in	
Humid ventilation: sterile aqua dest pierced	

Checklist Setup
Ventilator „Evita® 500“ (Dräger)
Filter ventilation

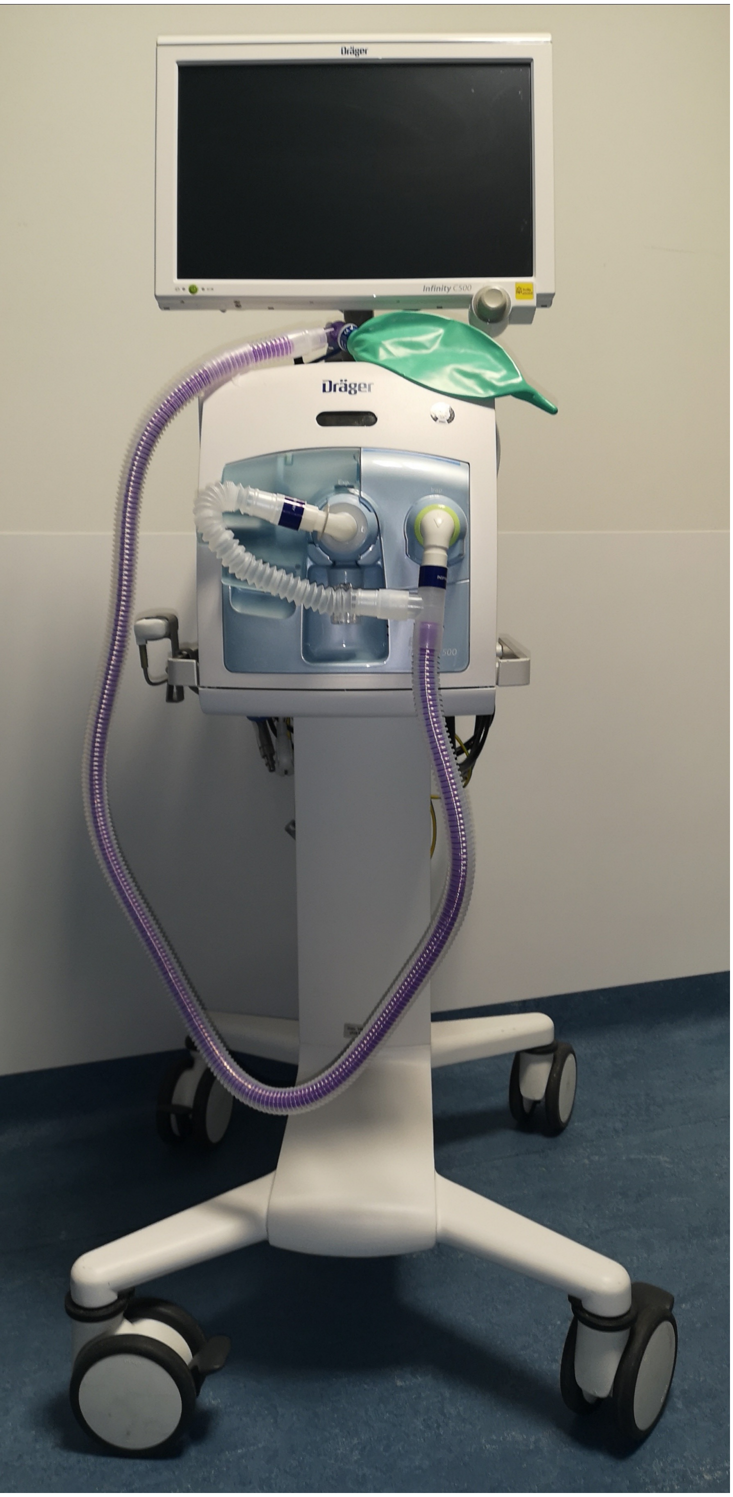

Hose system purple up to 15 kg with filter!

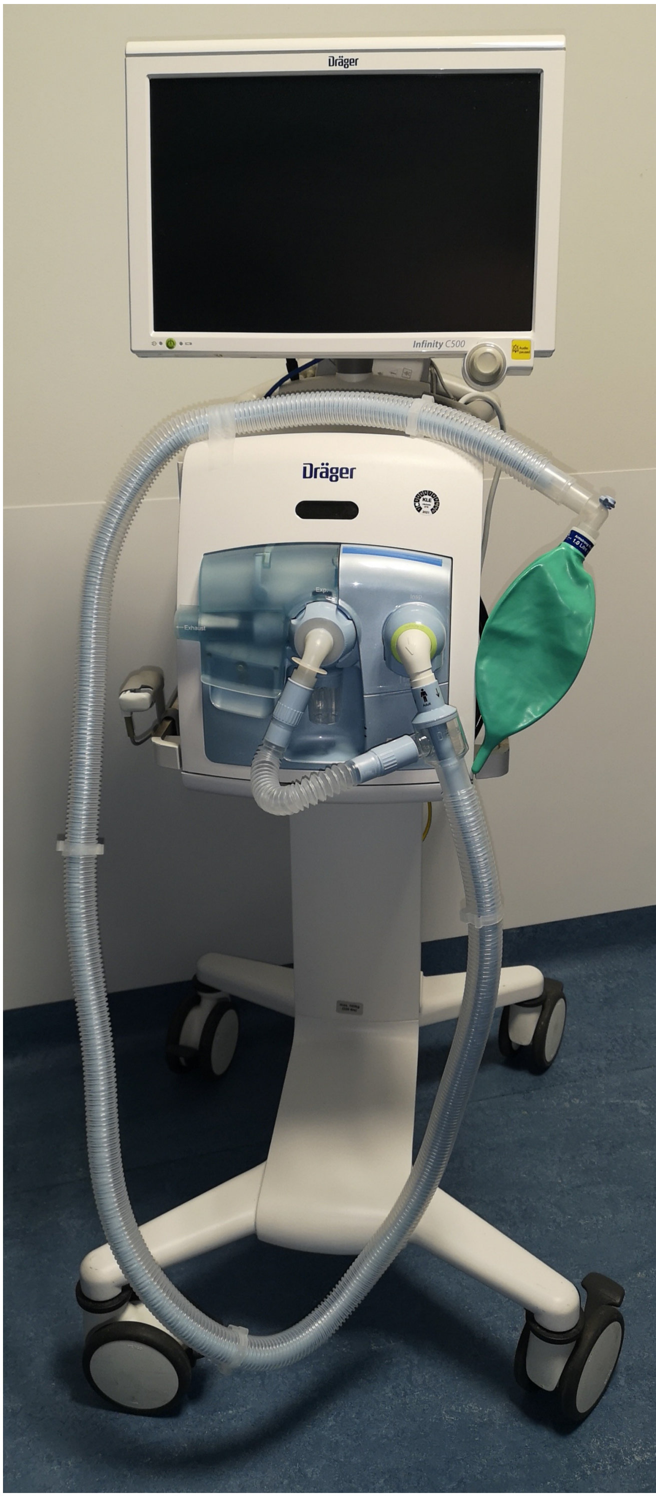

Hose system blue from 15 kg with filter!

Checklist Start-up
Ventilator „Evita® 500“ (Dräger)

Switch on ventilator and heating if necessary	
Carry out system check and breathing tube check	
Choice of ventilation mode (invasive ventilation) and category (child or adult)	
Setting the weight (over height for adults if necessary)	
Setting the ventilation parameters according to the expected patient	
Setting the alarm limits	
Display of at least the following values: VT/kg bw, MVe, respiratory rate, FiO2, Pmean, etCO2, leak, meaningful display of the measured values	

**Parameters**	**5 kg**	**10 kg**	**15 kg**	**20 kg**	**30 kg**	**40 kg**	**50 kg**	**60 kg**
PIP (cmH_2O)	15	15	15	15	15	15	15	15
PEEP (cmH_2O)	5	5	5	5	5	5	5	5
Frequency (/min)	35	30	25	20	18	15	13	12
T insp (sec)	0,45	0,6	0,7	0,8	1,0	1,1	1,2	1,3
MV high (l/min)	*1,5*	*2,0*	*3,0*	*3,5*	*3,9*	*5,2*	*6,5*	*7,8*
MV low (l/min)	*0,8*	*1,0*	*1,5*	*1,8*	*2,1*	*2,8*	*3,5*	*4,2*
VT high (ml)	*40*	*80*	*120*	*160*	*240*	*320*	*400*	*480*
VT low (ml)	*15*	*30*	*45*	*60*	*90*	*120*	*150*	*180*

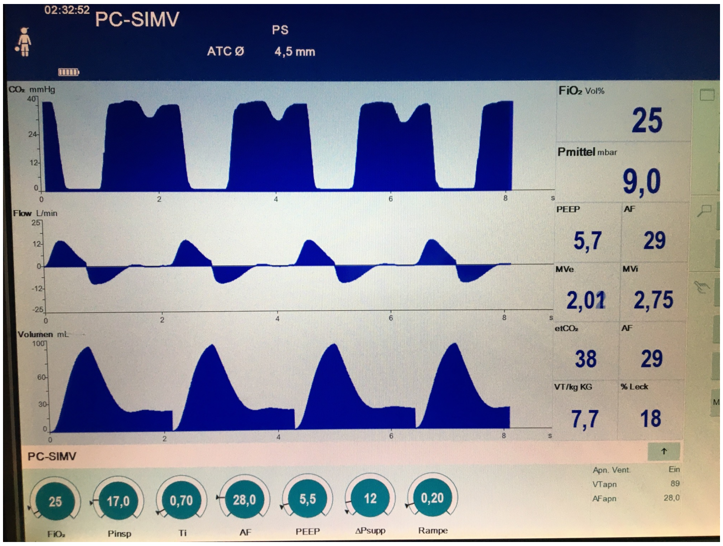

Weight-independent:
Ramp: 0,2 sec
Consider pressure support
(CAVE: pressure support as delta-P over PEEP)

Leak 30%
Apnoea 10 sec
Ppeak 20
Frequency: Default frequency + 10

Checklist Setup „Evita® 500“ with NO

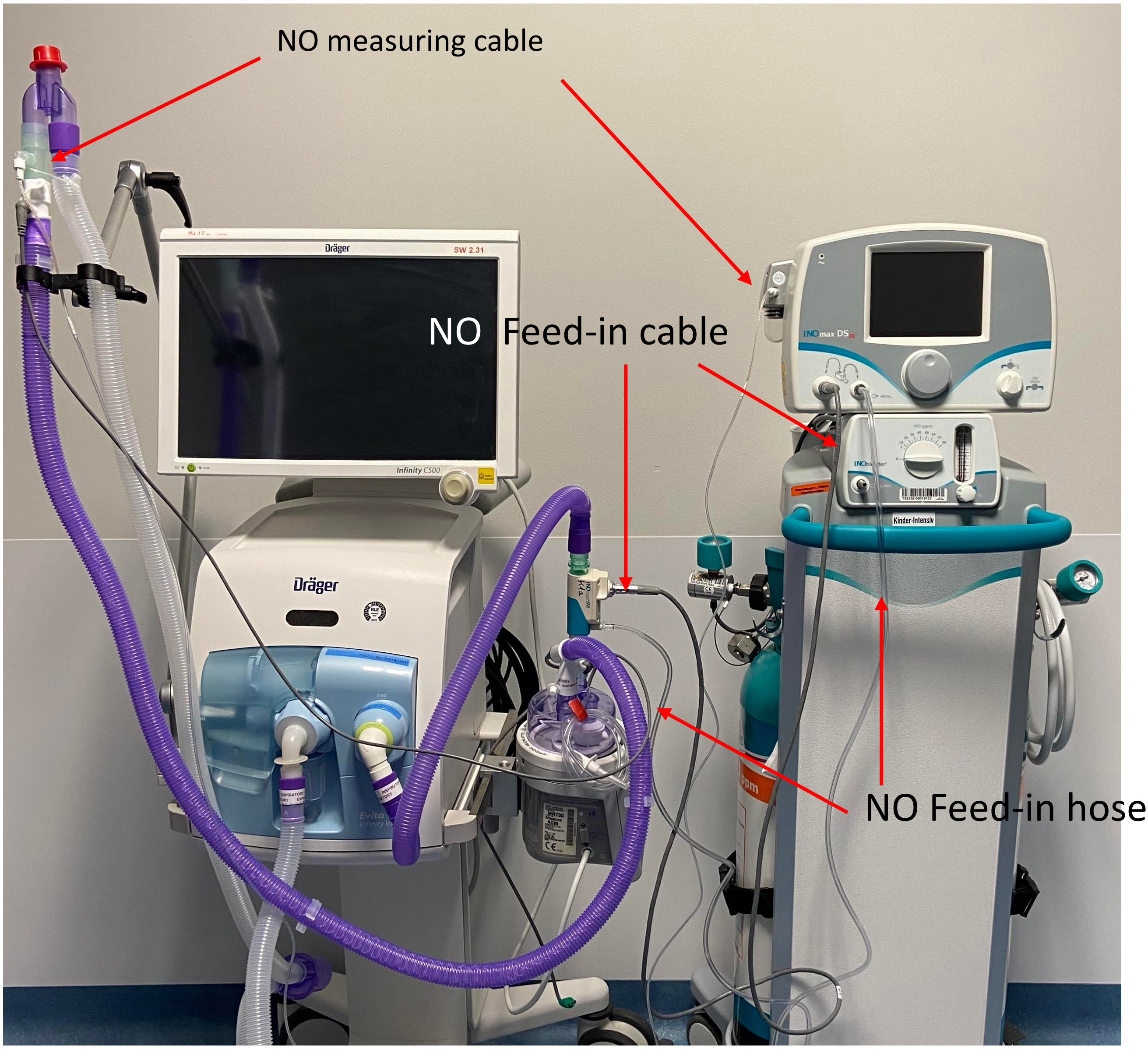

<u>Required adapters:</u><br>Feed-in: connector 22M/15F (close to the device), connector 22F/15M (close to the heating system)<br>Detector: connector 22M – 22F + 7,6 mm Port, Luer-Elbow-Clip	
Install the NO Feed-in module with cable and hose with adapter in the inspiration flow in front of the heating pot	
Install NO sensor cable with adapter in inspiratory flow close to patient	
