# Supplementary material for: Pilot study of an interprofessional pediatric mechanical ventilation educational initiative in two intensive care units
Source: BMC Med Educ. 2023 Aug 28;23:610. doi: 10.1186/s12909-023-04599-1 (PMC10463469; doi:10.1186/s12909-023-04599-1)
Supplement: Supplementary file 2 — Additional file 2: Supplementary Figure 1. The flowchart shows the number of pre-and post-intervention recruited participants and the statistical analyses performed. [file 12909_2023_4599_MOESM2_ESM.pdf]

# Recruitment of participants and analyses

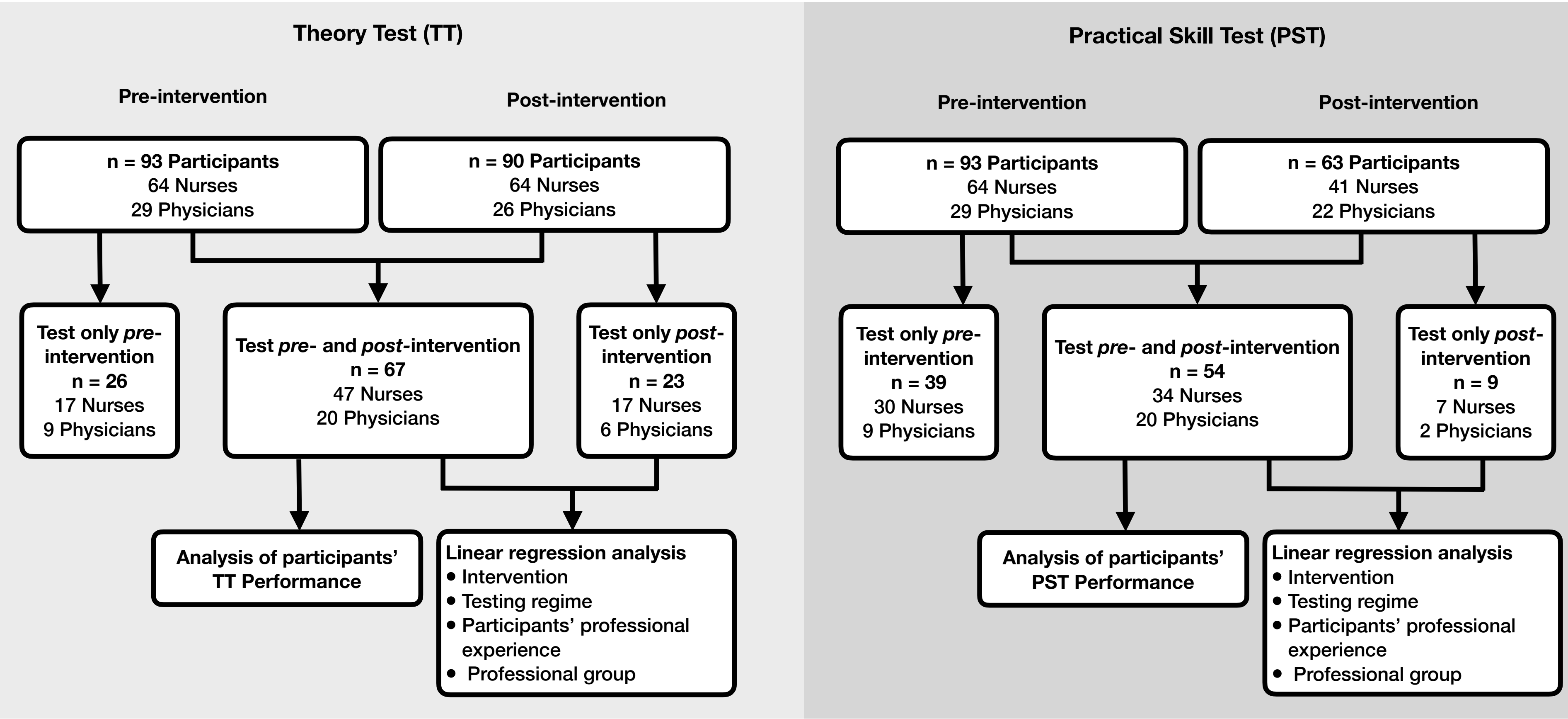


**Supplementary Figure 1:** The flowchart shows the number of pre-and post-intervention recruited participants and the statistical analyses performed.
