# Supplementary material for: Pilot study of an interprofessional pediatric mechanical ventilation educational initiative in two intensive care units
Source: BMC Med Educ. 2023 Aug 28;23:610. doi: 10.1186/s12909-023-04599-1 (PMC10463469; doi:10.1186/s12909-023-04599-1)
Supplement: Supplementary file 3 — Additional file 3: Supplementary Figure 2. Self-confidence of nurses (top) and physicians (bottom) before vs. after a pediatric mechanical ventilation education intervention. [file 12909_2023_4599_MOESM3_ESM.pdf]

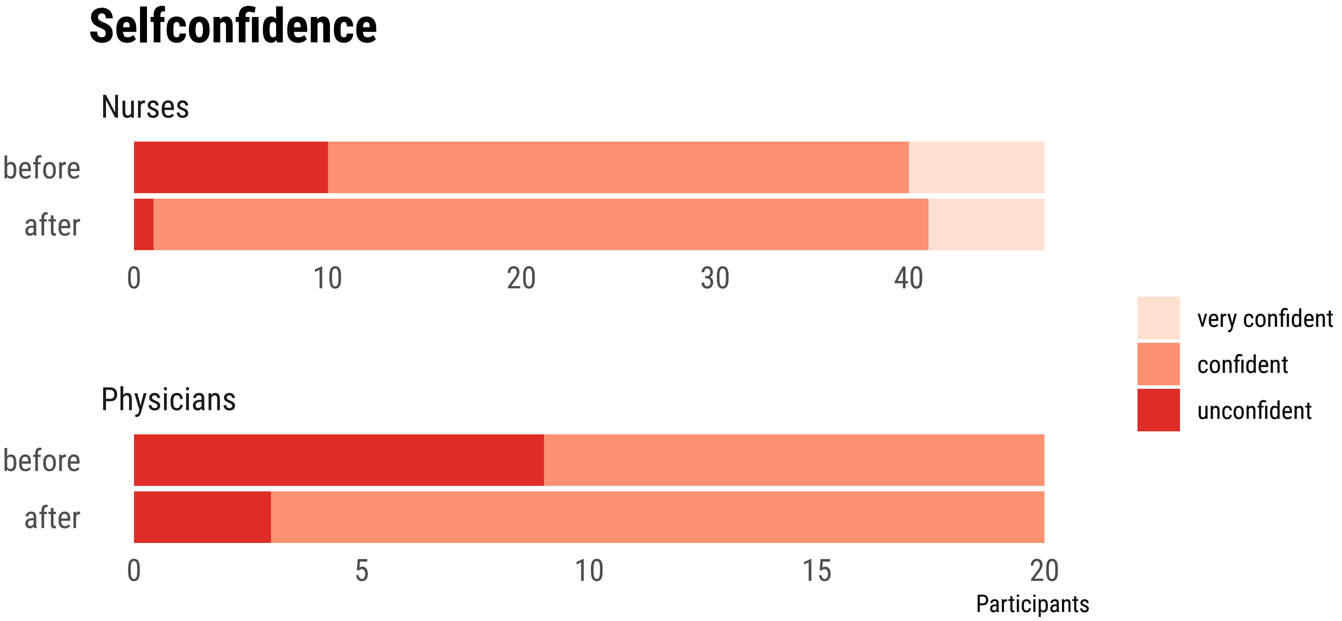


**Supplementary Figure 2:** Self-confidence of nurses (top) and physicians (bottom) before vs. after a pediatric mechanical ventilation education intervention.
