# Supplementary material for: Pilot study of an interprofessional pediatric mechanical ventilation educational initiative in two intensive care units
Source: BMC Med Educ. 2023 Aug 28;23:610. doi: 10.1186/s12909-023-04599-1 (PMC10463469; doi:10.1186/s12909-023-04599-1)
Supplement: Supplementary file 4 — Additional file 4: Supplementary Figure 3. A) Average performance of participants in the Theory Test Category and the Practical Skill Test Topic Category, categorized by their respective professional groups and experience. B) Detailed view of the performance related to each individual aspect of the practical skill test, as well as for the individual topics of the theory test, separately for the two occupational groups, nurses, and physicians. The dashed vertical red lines represent the average performance before the intervention, while the solid lines depict the performance after the intervention. The gray diamonds indicate the mean performance per topic or task of the tests before the intervention, and the black diamonds represent the mean performance after the intervention. iNO: Inhaled Nitric Oxide, HFOV: High Frequency Oscillatory Ventilation, DOPES: Acronym: Dislocation, Obstruction, Pneumothorax, Equipment, Stomach, FiO2: Fraction of Inspiratory Oxygen. [file 12909_2023_4599_MOESM4_ESM.pdf]

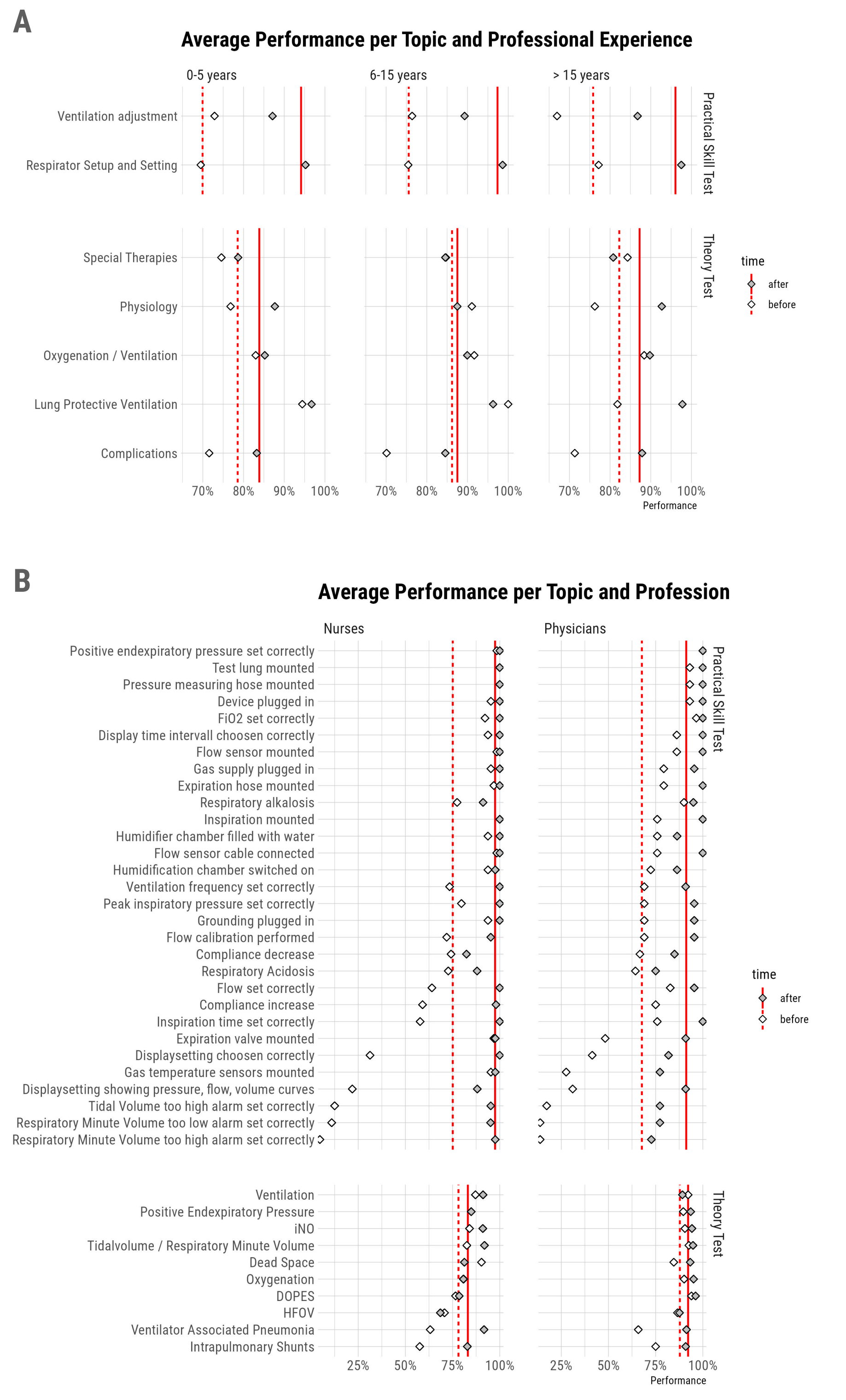
A
Average Performance per Topic and Professional Experience
0-5 years
6-15 years
> 15 years
Ventilation adjustment
Respirator Setup and Setting
Practical Skill Test
Special Therapies
Physiology
Oxygenation / Ventilation
Lung Protective Ventilation
Complications
Theory Test
time
after
before
70%
80%
90%
100%
Performance
B
Average Performance per Topic and Profession
Nurses
Physicians
Positive endexpiratory pressure set correctly
Test lung mounted
Pressure measuring hose mounted
Device plugged in
FiO2 set correctly
Display time intervall choosen correctly
Flow sensor mounted
Gas supply plugged in
Expiration hose mounted
Respiratory alkalosis
Inspiration mounted
Humidifier chamber filled with water
Flow sensor cable connected
Humidification chamber switched on
Ventilation frequency set correctly
Peak inspiratory pressure set correctly
Grounding plugged in
Flow calibration performed
Compliance decrease
Respiratory Acidosis
Flow set correctly
Compliance increase
Inspiration time set correctly
Expiration valve mounted
Displaysetting choosen correctly
Gas temperature sensors mounted
Displaysetting showing pressure, flow, volume curves
Tidal Volume too high alarm set correctly
Respiratory Minute Volume too low alarm set correctly
Respiratory Minute Volume too high alarm set correctly
Practical Skill Test
Ventilation
Positive Endexpiratory Pressure
iNO
Tidalvolume / Respiratory Minute Volume
Dead Space
Oxygenation
DOPES
HFOV
Ventilator Associated Pneumonia
Intrapulmonary Shunts
Theory Test
time
after
before
25%
50%
75%
100%
Performance
