# Supplementary material for: Pilot study of an interprofessional pediatric mechanical ventilation educational initiative in two intensive care units
Source: BMC Med Educ. 2023 Aug 28;23:610. doi: 10.1186/s12909-023-04599-1 (PMC10463469; doi:10.1186/s12909-023-04599-1)
Supplement: Supplementary file 5 — Additional file 5: sTable 1. Mixed-effects model for treatment goal compliance. [file 12909_2023_4599_MOESM5_ESM.docx]

| **sTable 1: Mixed-effects model for treatment goal compliance** | | | |
| --- | --- | --- | --- |
| *Predictors* | *Estimates* | *CI* | *p* |
| Intervention (before) | -0.07 | -0.09 – -0.05 | **<0.001** |
| Lenght of stay (days) | -0.00 | -0.00 – 0.00 | 0.992 |
| Patient category (neonate) | 0.02 | -0.01 – 0.06 | 0.138 |
| ICU (PICU) | -0.04 | -0.08 – -0.00 | **0.027** |
| **Random Effects** | | | |
| σ^2^ | 0.01 | | |
| τ_00 Patient ID_ | 0.00 | | |
| ICC | 0.13 | | |
| N _Patient ID_ | 213 | | |
| Observations | 662 | | |
| Marginal R^2^ / Conditional R^2^ | 0.141 / 0.252 | | |

ICU: intensive care unit, ICC: intraclass correlation coefficient.
